# Modeling the Effects of the Environment and the Host Plant on the Ripe Rot of Grapes, Caused by the *Colletotrichum* Species

**DOI:** 10.3390/plants10112288

**Published:** 2021-10-25

**Authors:** Tao Ji, Irene Salotti, Chaoyang Dong, Ming Li, Vittorio Rossi

**Affiliations:** 1 Department of Sustainable Crop Production (DI.PRO.VES.), Università Cattolica del Sacro Cuore, Via E. Parmense 84, 29122 Piacenza, Italy; tao.ji@unicatt.it (T.J.); irene.salotti1@unicatt.it (I.S.); 2 Tianjin Climate Center, Tianjin 300074, China; dongchaoyang0606@126.com; 3 National Engineering Research Center for Information Technology in Agriculture (NERCITA) and Information Technology Research Center, Beijing Academy of Agriculture and Forestry Sciences, Beijing 100097, China; lim@nercita.org.cn

**Keywords:** life cycle, epidemiology, disease modeling, model validation

## Abstract

Ripe rot caused by *Colletotrichum* spp. is a serious threat in many vineyards, and its control relies mainly on the repeated use of fungicides. A mechanistic, dynamic model for the prediction of grape ripe rot epidemics was developed by using information and data from a systematic literature review. The model accounts for (i) the production and maturation of the primary inoculum; (ii) the infection caused by the primary inoculum; (iii) the production of a secondary inoculum; and (iv) the infection caused by the secondary inoculum. The model was validated in 19 epidemics (vineyard × year combinations) between 1980 and 2014 in China, Japan, and the USA. The observed disease incidence was correlated with the number of infection events predicted by the model and their severity (ρ = 0.878 and 0.533, respectively, n = 37, *p* ≤ 0.001). The model also accurately predicted the disease severity progress during the season, with a concordance correlation coefficient of 0.975 between the observed and predicted data. Overall, the model provided an accurate description of the grape ripe rot system, as well as reliable predictions of infection events and of disease progress during the season. The model increases our understanding of ripe rot epidemics in vineyards and will help guide disease control. By using the model, growers can schedule fungicides based on the risk of infection rather than on a seasonal spray calendar.

## 1. Introduction

Ripe rot of grapes is an important disease that not only can cause severe yield losses and deterioration of grape vines, but also reduces grape and wine quality, including color and flavor. In addition, the disease increases volatile acidity and the contents of residual sugar, glycerol, gluconic acid, and malic acid, resulting in a bitter off-flavor for the fruit and wine [1,2]. Ripe rot was first reported in the USA [3] and is currently present wherever host species (*V. labrusca*, *V. vinifera*, and *V. rotundifolia* [4]) are grown. The disease is caused by *Colletotrichum* species belonging to the complex *C. gloeosporioides* and *C. acutatum* [2,5,6,7,8,9,10,11,12,13,14,15,16].

Ripe rot can affect grapevine leaves and stems but is most important on clusters [4,6]. The typical disease symptoms usually appear on ripening berries after véraison and near harvest. Affected berries initially develop circular, reddish-brown spots on their skin, which then enlarge in concentric lesions that can occupy the whole berry and that develop acervuli (small asexual fruiting bodies) with orange-pink spore masses that erupt through the epidermis. Berries soften and shrivel, and may remain on the rachis or drop to the soil [4].

The control of ripe rot relies mainly on the repeated use of fungicides throughout the growing season. Lime sulfur is applied before bud break to reduce the overwintered inoculum [6]; strobilurin fungicides are recommended at flowering [17], and protective broad-spectrum fungicides such as captafol, captan, maneb, or benomyl are repeatedly applied from “small green fruits” to “fruit turning bronze” at 2-week intervals [18]. Such an intensive fungicide calendar is not economically or ecologically sound and may result in needless sprays. In the context of a more sustainable use of pesticides [19], there is an increasing interest in moving from calendar-based to infection-risk-based fungicide applications [20,21].

To reduce the risk of unnecessary sprays for disease control in vineyards, researchers have developed process-based mathematical models [22,23,24,25,26,27]. To our knowledge, there are only few reports on mathematical models for ripe rot of grapes. Yun and Park [28] developed an infection model based on temperature and wetness duration, and Park et al. [29] further developed a forecasting system based on this model and tested it in Korea [29]. This forecasting system, however, considers only the influence of temperature and wetness duration on a single component of ripe rot epidemiology (i.e., germination of conidia). Then, Park’s forecasting system fails to consider the production and dispersal of inoculum and other epidemiological parameters. Therefore, a model incorporating all of the key epidemiological components leading to *Colletotrichum* infection is still lacking.

The aim of the present study was to develop a new, process-based dynamic model that can quantitatively evaluate the effect of environmental conditions on the life cycle of *Colletotrichum* species causing ripe rot, and that can thereby predict the risk of infection in vineyards. For this purpose, we (i) conducted a systematic literature search to retrieve the current knowledge on grape ripe rot; (ii) mobilized this knowledge to develop a conceptual model of the *Colletotrichum* species life cycle based on systems analysis; (iii) developed the mathematical equations describing the system both quantitatively and dynamically; (iv) evaluated the capability of the model to represent the real system; and finally (v) identified gaps in our knowledge that require further research.

## 2. Results

### 2.1. Model Description

**Figure 1:**
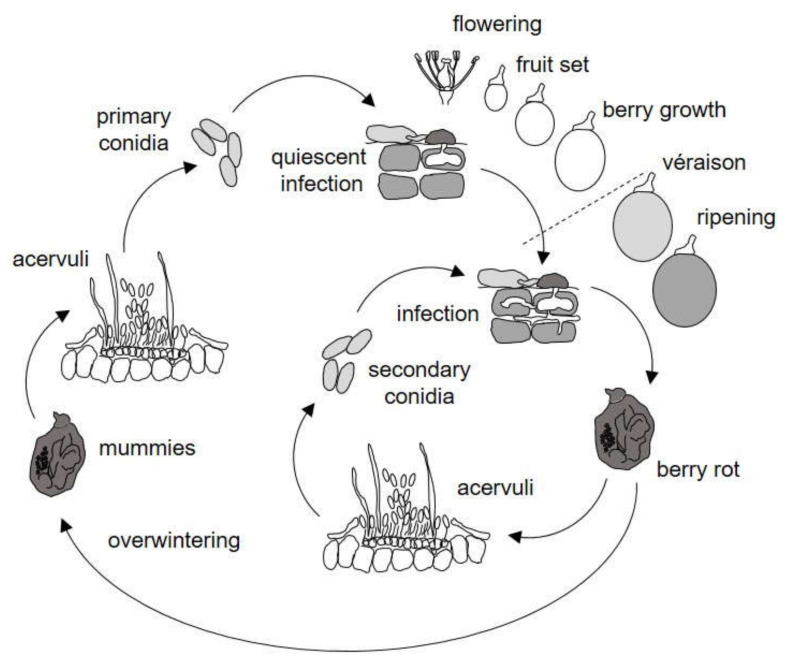
Life cycle of *Colletotrichum* spp. on grapes as considered in the model.

**Figure 2:**
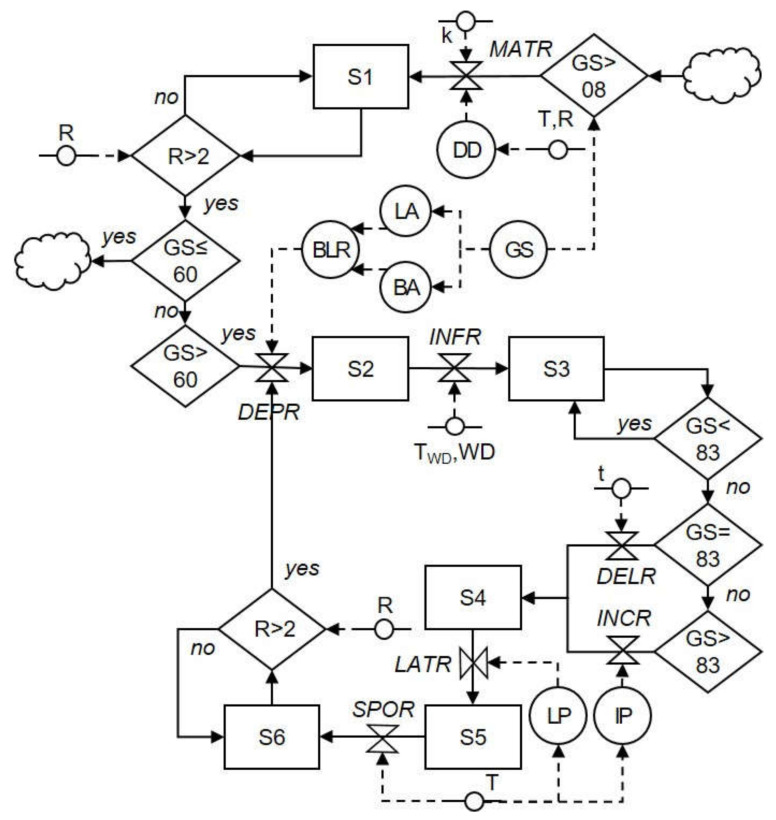
Relational diagram of the processes leading to berry infection by *Colletotrichum* spp. on grapes, based on the current knowledge from the literature. Boxes represent state variables; arrows show the flux and direction of data from one state variable to another; diamonds contain conditions resulting in a flux; valves indicate rate variables regulating a flux; segments with circles indicate external variables; circles indicate auxiliary variables; broken arrows link external or auxiliary variables to diamonds or throttles that they influence; clouds indicate state variables which enter or exit from the system (not quantified). All variables are listed in Table 1. Based on the model evaluations of this work, the switch related to rain for the dispersal of secondary conidia (S6) was modified as follows: R > 0.

**Figure 3:**
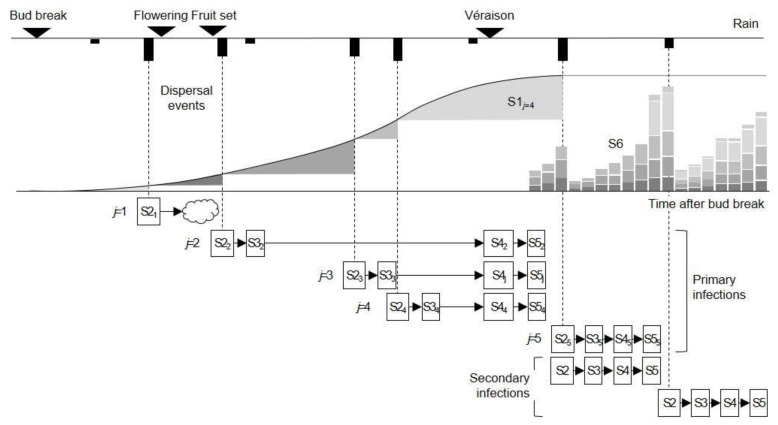
Simplified representation of how the model that predicts berry infection by *Colletotrichum* spp. on grapes works based on the diagram in Figure 2. The primary conidia develop over the season, starting from the vines’ bud break (the S-shaped line that becomes dotted when there are no more primary conidia) and, at each dispersal event triggered by a rain >2 mm/h (dotted vertical lines), a cohort *j* of mature conidia (S1*_j_*; gray areas) is deposited on flowers (during flowering) or berries (from fruit set on) (S2). These conidia cause infections (S3), which remain quiescent until véraison, cause berry rot (S4), and become fertile (S5), thus producing secondary conidia. Secondary conidia (S6) accumulate on berries and start new, secondary infection cycles at each dispersal event. All variables are listed in Table 1.

The life cycle of *Colletotrichum* spp. on grapes as deduced from the literature is shown in Figure 1. The relational diagram of the processes leading to infection by *Colletotrichum* spp. and the consequent ripe rot epidemics on grapes is shown in Figure 2; abbreviations are explained in Table 1. The relational diagram describes the main stages of the *Colletotrichum* spp. life cycle, including the following: (i) production and maturation of primary inoculum, (ii) infection caused by primary inoculum, (iii) production of secondary inoculum, and (iv) infection caused by secondary inoculum. A simplified representation of how the model works is shown in Figure 3.

Our “system of interest” consisted of a single grape plant surrounded by similar grape plants in terms of size, growth, and development, and by the disease caused by *Colletotrichum* spp. on clusters. The model is intended to support disease control by preventing infection. For this reason, the model does not simulate the dynamics of rotting berries during the season, but instead simulates the periods in which there are infections (primary and secondary) and the relative severity of these infections. The relative severity for primary infections is expressed as the proportion of the dose of the conidia produced by affected and mummified clusters that overwintered in the vineyard and that mature, disperse, and settle on flowers and berries, and then infect and finally cause disease symptoms. The relative severity for secondary infections is expressed as the proportion of secondary conidia produced by the infecting conidia that disperse and settle on berries, and then infect and finally cause disease symptoms.

#### 2.1.1. Production and Maturation of Primary Inoculum

The model assumes that there is a potential dose of conidia from the overwintered inoculum sources in the vineyard (represented by the a 0–1 parameter, k) that will develop during the season from affected cluster mummies (berries, pedicels, and rachises) that overwintered in the vineyard, starting from bud break (the growth stage GS = 08 according to the scale of BBCH [30]).

These conidia become progressively available during the season at a maturation rate (*MATR*) that depends on the temperature (T), and more specifically on the degree-days (DD) accumulated from bud break when there is sufficient moisture provided by rain (R). These conidia enter into the 1^st^ state variable of the model, S1. On any i^th^ day after bud break, the model calculates the proportion of conidia entering S1 through a conidial maturation rate, *MATR*_i_, which is calculated as the first-order derivative of the following Equation:

Y_i_ = k (1 − exp( − ((0.0043 DD_i_)^1.5656^))) (1)

where Y is the cumulative proportion of mature, primary conidia produced on day i, and DD is the cumulative degree-days accumulated from bud break on those days when T > 5 °C and rain > 1 mm. The estimates and standard errors of the equation parameters were 0.0043 ± 0.0001 and 1.5656 ± 0.0952, respectively. Equation (1) was developed by using data of Fukaya [31] and Daykin and Miholland [18], with R² = 0.807 (see Figure S1 in the Supplementary Materials for further details).

#### 2.1.2. Spore Dispersal

Mature conidia in S1 are dispersed by a splashing rain >2 mm/h (see the Supplementary Materials) and are deposited on flowers or berries (the 2nd state variable, S2), from flowering onward (i.e., GS > 60); because the conidia that disperse up to flowering do not cause infection, they are considered “lost” (the cloud symbol). In the model, the conidia entering S2 form a cohort *j* of different size that initiates a primary infection cycle, until the production of the secondary inoculum; the size of each *j*^th^ cohort of conidia depends on S2 at the time of dispersal, as shown in Figure 3. The model then considers as many primary infection cycles as there are dispersal events of primary inoculum.

The model assumes that the dose of conidia, which is deposited on flower or cluster surfaces, depends on the leaf and berry area (the auxiliary variables LA and BA, respectively) and a deposition rate (*DEPR*) that in turn depends on a berry-to-leaf ratio (the auxiliary variable BLR, calculated as BA/(LA+BA)), both of which depend on the GS (see Figure S2 in the Supplementary Materials). Therefore,

S2*_j_*_i_ = S1_i_ BLR_i_ (2)

#### 2.1.3. Primary Infection Events

Conidia in each *j*^th^ cohort of S2 cause infection on flowers and berries via an infection rate (*INFR*), which depends on the wetness duration (WD, in h) and the temperature during the wet period (T_WD_, in °C). These conidia thereby generate the 3rd state variable (S3), i.e., the number of conidia per plant causing infections (Figure 2). Therefore,

S3*_j_*_i_ = S2*_j_*_i_
*INFR_j_*_i_ (3)

The infection rate is calculated as the product of two equations that account for the effect of temperature, *f*(T), and the wetness duration, *f*(WD). The first equation concerns temperature:

*f*(T) = ((1.453 + 1)/1.453)1.453^(1/(1.453 + 1))^ (4)

exp((T_WD_ − 27.686)0.520/(1.453 + 1))/(1 + exp((T_WD_ − 27.686)0.520))

where T_WD_ is the mean temperature during the wet period (°C). The estimates and standard errors of the equation parameters were 27.686 ± 1.843, 0.520 ± 0.106, and 1.453 ± 1.100. Equation (4) was developed by using the data of Steel et al. [17] and Yun and Park [28], with R² = 0.876 (see Figure S3 in the Supplementary Materials). The second equation concerns wetness duration:

*f*(WD) = 1 − exp(− ((0.065WD)^3.321^)) (5)

where WD is the duration of the wet period (i.e., the number of hours with uninterrupted leaf wetness or interrupted by a maximum of 3 h). The estimates and standard errors of the equation parameters were 0.065 ± 0.002 and 3.321 ± 0.498. Equation (5) was developed by using the data of Greer et al. [32] and Yun and Park [28], with R² = 0.945 (see Figure S4 in the Supplementary Materials).

The model considers that fruit is equally susceptible at any time during the season [4], so that there are no modifiers for the infection severity based on the growth stage of clusters; infections, however, may be quiescent or active based on the growth stage [18,33].

The model assumes that fruit infections occurring before véraison remain quiescent until véraison, when they progressively result in berry rot (the 4th state variable, S4, namely the conidia leading to infection and result in visible disease symptoms, i.e., berry rot) starting from GS = 83 (véraison), at a rate (*DELR*) that depends on the time (t, in days), so that *DELR* = 1/t. Because we did not find data on the time required by berries with quiescent infections to develop disease symptoms after véraison, t is kept constant at 5 days in the model. Therefore,

S4*_j_*_i_ = S3*_j_*_i_ *DELR*_i_ (6)

The model considers that infections occurring after véraison also result in berry rot (S4) after an incubation period (IP, in days) that depends on the temperature, at an incubation rate (*INCR*), so that *INCR* = 1/IP. We did not find precise data on the incubation length as affected by temperature; therefore, the IP is kept constant at 3 days, which is the time when 50% of the grape berries that have been artificially inoculated with conidia of *C. acutatum* or *C. gloeosporioides* and then incubated at 27 °C and 100% RH showed disease symptoms in Steel at al. [34]. Therefore,

S4_i_ = S3_i_ *INCR*_i_ (7)

The relative severity of primary infection events was then calculated as S4*_j_*/k.

#### 2.1.4. Production of Secondary Inoculum

The model considers that the colonies in S4 become fertile (the 5th state variable, S5) at a rate that depends on the latency period (the auxiliary variable LP), which in turn depends on the temperature, so that *LATR* = 1/LP. We did not find data on the length of latency as affected by temperature; therefore, the LP is kept constant at 5 days [35], and S5_i_ is calculated as follows:

S5_i_ = S4_i_ *LATR*_i_ (8)

Colonies in S5 then produce secondary conidia at a sporulation rate (*SPOR*). The secondary conidia accumulate in the 6th state variable, S6. *SPOR* is calculated on each i^th^ day as a function of temperature, as follows:

*SPOR*_i_ = (6.750Teq_i_^2.000^ (1 − Teq_i_))^1.128^ (9)

S6_i_ = S5_i_ *SPOR_i_* (10)

where Teq is an equivalent of temperature calculated as Teq = (T–Tmin)/(Tmax–Tmin); with T = mean temperature during the incubation time (°C), Tmin = minimum temperature for sporulation (4 °C), and Tmax = maximum temperature for sporulation (36 °C). The estimates and standard errors of the equation parameters were 6.750 ± 0.827, 2.000 ± 0.264, and 1.128 ± 0.323, respectively. Equation (9) was developed by using data of Es-Soufi et al. [36], Everett et al. [37], Fernando et al. [38], King et al. [39], Liu et al. [40], Fitzell and Peak [41], Mello et al. [42], Pandey et al. [43], Wastie [44], Wang et al. [45], and Veloso et al. [46], with R² = 0.830 (see Figure S5 in the Supplementary Materials).

#### 2.1.5. Secondary Infection Events

As was the case for primary infections, the model assumes that on any day i^th^ with R > 2 mm/hour, all of the conidia accumulated in S6 after the previous dispersal event (these conidia can be produced by different *j*^th^ cohorts of primary conidia and different secondary infection events; see Figure 2) can be deposited on berries at a deposition rate *DEPR* and cause infection at an infection rate *INFR*, which are calculated as described before for primary infections.

As was the case for primary infection events, the relative severity of secondary infection events was then calculated as S4/k.

### 2.2. Model Evaluation

#### 2.2.1. Predictions and Disease Incidence

**Figure 4:**
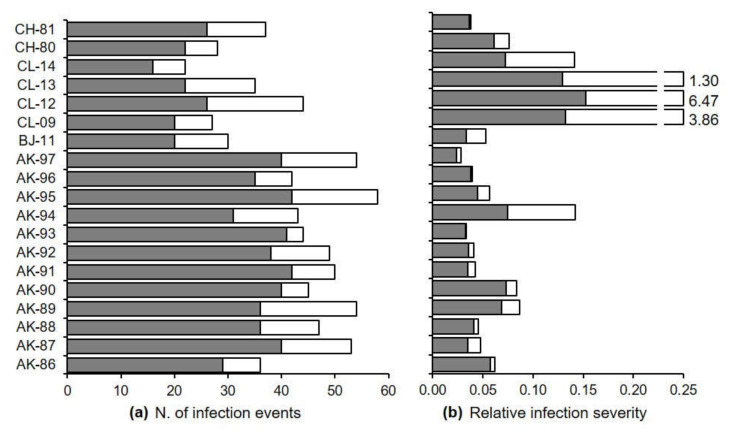
Number (**a**) and severity (**b**) of primary (gray bars) and secondary (white bars) infection events predicted by the model in the 19 epidemics of Table 2.

The model predicted variable numbers of infection events for the 19 epidemics described in Table 2, ranging from a minimum of 22 (for CL-14) to a maximum of 58 (for AK-95) in the period between flowering and harvest; most of these infection events (59 to 93% of the total events) were caused by primary conidia (Figure 4a). Predictions of accumulated infection severities were also variable, with a minimum of 0.03 (AK-97) and a maximum of 6.47 (CL-12); the contribution of secondary infections was generally lower than that of primary infection, except for epidemics CL-09, CL-12, and CL-13 (Figure 4b).

**Figure 5:**
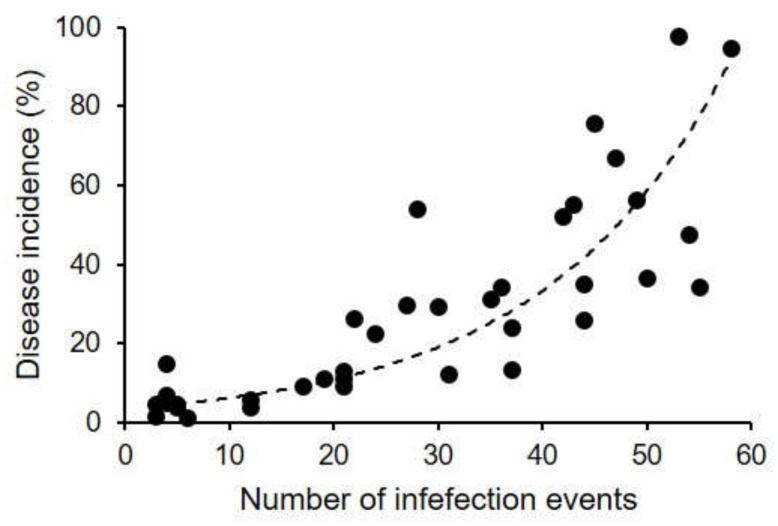
Relationship between the observed ripe rot incidence at harvest (as % of affected clusters or berries, depending on the epidemic; see Table 2) and number of infection events predicted by the model. The dotted line represents the regression of these data by an exponential equation, with R² = 0.757 and n = 37.

The disease incidence observed in vineyards was significantly correlated with all of the model predictions. The Spearman’s correlation coefficient (ρ) was 0.878, 0.865, and 0.801 for total, primary, and secondary infection events, respectively (n = 37, *p* < 0.001); ρ was 0.533 (*p* = 0.001), 0.402 (*p* = 0.014), and 0.707 (*p* < 0.001) for the accumulated infection severities for total, primary, and secondary infections, respectively. The observed disease incidence (Y) increased exponentially as the number of total predicted infections increased (X) based on the following equation: Y = 3.5328 exp(0.0562 X), R^2^ = 0.757 (Figure 5). These results collectively indicated that the model was able to interpret the disease pressure in the considered cases, and that infection predictions for both primary and secondary cycles helped explain the epidemics.

#### 2.2.2. Prediction of Infection Events and Disease Progress

**Figure 6:**
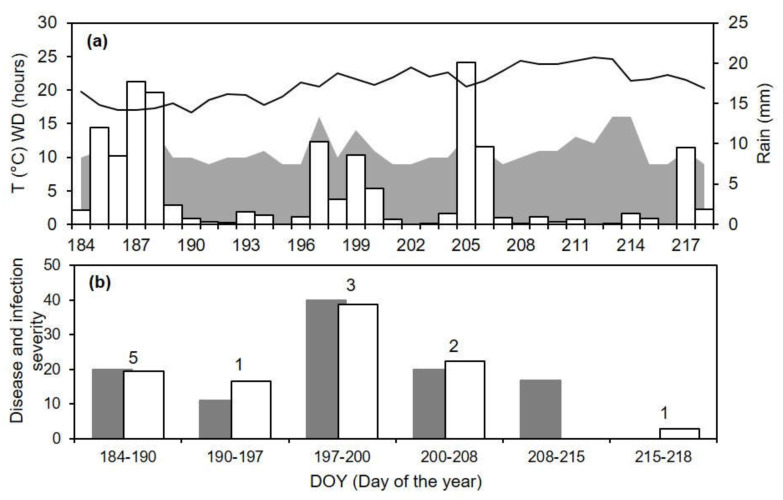
Comparison between model output and ripe rot severity for epidemic AK-96 (see Table 2 for epidemic characteristics). (**a**) Temperature (T, line), wetness duration (WD, gray area), and rain (bars). (**b**) Observed increases of ripe rot severity (as the % of cluster surface with disease symptoms; gray bars) and predicted accumulated infection severity (white bars) at different time intervals; values above the bars indicate the number of predicted infection events. For the clarification of the comparisons of the model predictions with the observed data, infection severity was rescaled to 100% (where 100 is the total of infection severities during the study period).

When the occurrences of infection events predicted by the model (as predicted, P+, or not predicted, P−) were compared with the observations of disease symptoms in clusters exposed to the natural inoculum in vineyards (as observed, O+, or not observed, O−) for AK-96 (Figure 6), four of six exposure periods were correctly predicted by the model. Specifically, the clusters exposed between DOY 184 and 206 showed a 11.1 to 40.0% disease severity at ripening (DOY 266), and the model predicted the occurrence of infections, with variable severity (Figure 6b). On the contrary, between DOY 208 and 215, the model missed the real infection (which resulted in a 16.7% disease severity at ripening), because it did not predict the dispersal of conidia. Even though the period was rainy (Figure 6a), the rain intensity was ≤0.9 mm/h, which was lower than the threshold of >2 mm/h that triggers conidial dispersal in the model. Clusters exposed between DOY 215 and 218 did not show the disease, but the model predicted a single infection event with low infection severity (Figure 6b).

**Figure 7:**
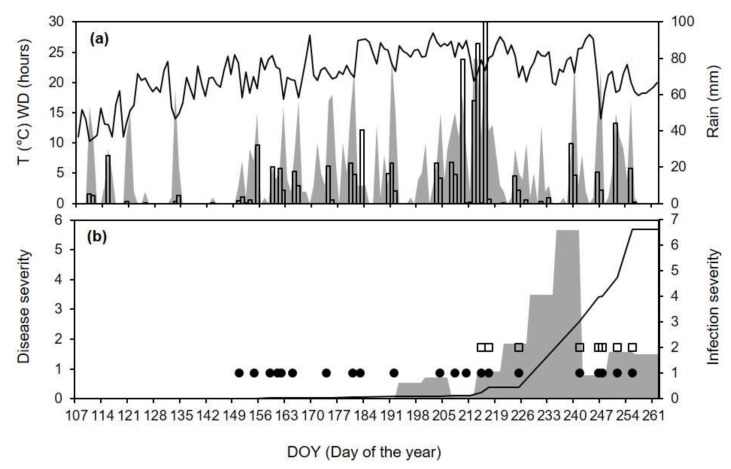
Comparison between model output and ripe rot severity for epidemic CL-12 (see Table 2 for epidemic characteristics). (**a**) Temperature (T, line), wetness duration (WD, gray area), and rain (bars). (**b**) Weekly increases in observed ripe rot severity (as the % of cluster surface with disease symptoms; gray area), days in which the model predicted primary (black dots) and secondary (white squares) infection events, and accumulated infection severity as predicted by the model (line).

For CL-12, the disease was first observed in mid-July (exactly on DOY 199), and the disease severity then increased by <2% per week until DOY 220, and then by 3.5% (on DOY 234) and 5.7% (on DOY 241); the disease severity increased by <2% in the two following weeks, until DOY 262 (Figure 7b). The model predicted 11 primary infection events between DOY 151 and 183, with very low severity (Figure 7b), which may have caused quiescent infections. In the period when the disease was observed in the vineyard, the model predicted 12 infection events (between DOY 192 and 255), and the last 8 events included both primary and secondary infections, which caused an increase in the accumulated disease severity (Figure 7b). The comparison O+|O− vs. P+|P− showed that there were 9 (of 11) P+O+ cases. In 1 case, the model failed to predict a real disease increase (i.e., P−O+) between DOY 235 and 241, which was the most severe of the season (Figure 7b). Before this time period, there were only two rainy days: on DOY 231, there was 1.2 mm of rain in 4 h around noon, with WD = 13 h and T_WD_ = 22.9 °C; on DOY 233, there was 3.1 mm of rain in 3 h in late afternoon, but the WD was only 3 h (Figure 7a). The model did not predict the infection on these days because the rain intensity was always <2 mm/h (the model threshold). There were no cases with P−|O− or P+|O−.

**Figure 8:**
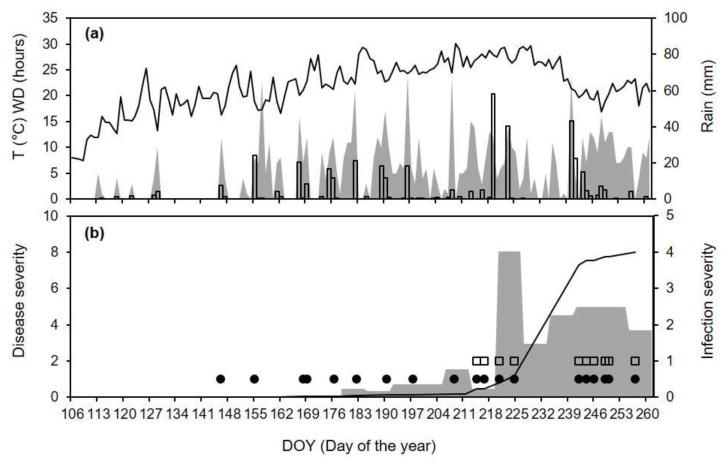
Comparison between model output and ripe rot severity at epidemic CL-13 (see Table 2 for epidemic characteristics). (**a**) Temperature (T, line), wetness duration (WD, gray area), and rain (bars). (**b**) Weekly increases in observed ripe rot severity (as the % of cluster surface with disease symptoms; gray area), days in which the model predicted primary (black dots) and secondary (white squares) infection events, and accumulated infection severity as predicted by the model (line).

For CL-13, the disease was first observed in early July (exactly on DOY 184), and disease severity subsequently increased by <2% per week until DOY 219. Between DOY 220 and 226, disease severity increased by 8.1% and subsequently continued to increase by 3.0 to 5.0% until DOY 261 (Figure 8b). The model predicted 15 days with infection between DOY 176 and 256, 10 of them with both primary and secondary infections, which caused a rapid increase of the predicted infection severity starting from DOY 214 (Figure 8b). The model correctly predicted 11 of 12 cases in which there was a disease increase in the vineyard (P+|O+). Between DOY 234 and 240, the observed disease severity increased by 4.5%, but the model did not predict infections (P−|O+) between DOY 225 and 240 (Figure 8b). On these days, there was only a 2-h rain period (0.2 mm in total) on the night of DOY 227 (Figure 8a), with WD = 6 h and T_WD_ = 26.9 °C; these conditions were insufficient to trigger spore dispersal in the model. As for CL-12, CL-13 had no cases with P−|O− or P+|O−.

**Figure 9:**
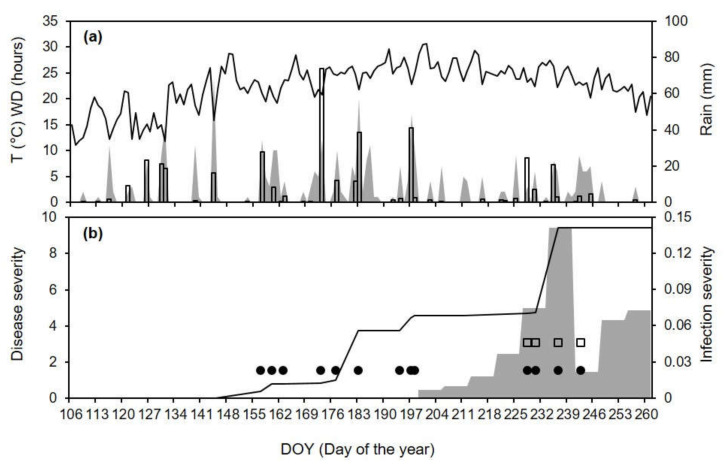
Comparison between model output and ripe rot severity at epidemic CL-14 (see Table 2 for epidemic characteristics). (**a**) Temperature (T, line), wetness duration (WD, gray area), and rain (bars). (**b**) Weekly increases in observed ripe rot severity (as the % of cluster surface with disease symptoms; gray area), days in which the model predicted primary (black dots) and secondary (white squares) infection events, and accumulated infection severity as predicted by the model (line).

For CL-14, the disease was first observed on the last days of July (exactly on DOY 205) just after the start of véraison of berries, and lightly increased for 3 weeks; disease severity then increased by 5.0 and 9.4% between DOY 227 and 233, and DOY 234 and 240, respectively. Afterward, the disease continued to develop, but with low severity until DOY 261 (Figure 9b). The model predicted nine primary infections before the onset of disease symptoms, with a high infection severity between DOY 183 and 198 (Figure 9b), when the weather was rainy and moist (Figure 9a). From DOY 199 and 227, no infections were predicted by the model because there were only light rains (7.9 mm in total, on 6 days, with a maximum intensity of 1.2 mm/h), and the weather was generally dry (a total of 38 h of wetness over the entire period) (Figure 9a); as indicated earlier in this paragraph, however, an increase in disease severity was observed. Four infection events (caused by both primary and secondary conidia) were then predicted during the period of rapid disease growth but not in the last phase of the epidemic (Figure 9b), when the weather was dry again (Figure 9a). Overall, there were 4 P+|O+ and 5 P−|O+ cases; as for CL-12 and CL-13, CL-14 had no cases with P−|O− or P+|O−.

Considering all four epidemics (AK-96, CL-12, CL-13, and CL-14), the model correctly predicted 28 of 36 cases in which an increase in disease was observed (i.e., TPP = 0.78). The eight cases in which the model did not predict real disease growth (i.e., FNP = 0.28) accounted for 21.5% of the total disease severity; all of these errors were related to light rains that did not trigger the dispersal of conidia in the model. Because our dataset included only one case in which there was no disease, we were not able to test the model for its ability to correctly predict no infection (P−|O−).

**Figure 10:**
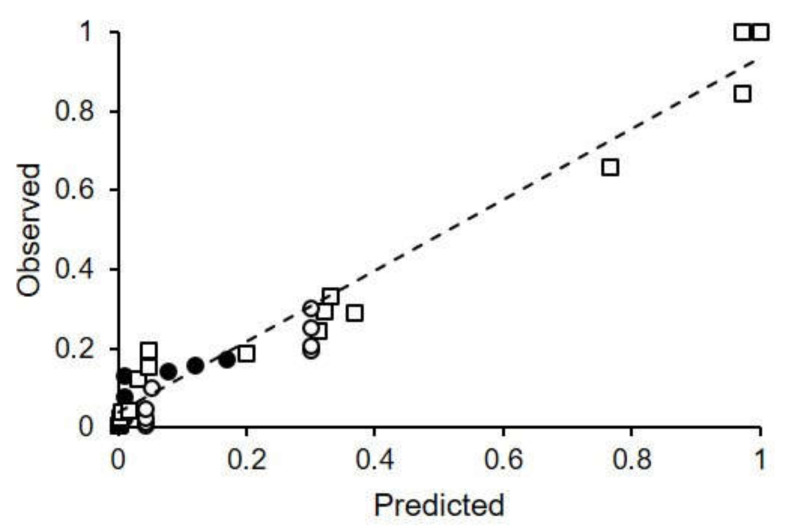
Plot of ripe rot severity observed in four epidemics (CL-12, black circles; CL-13, black squares; CL-14, white circles; and AK-96, white squares; see Table 2 for epidemic characteristics) vs. infection severity predicted by the model. The dotted line represents the linear regression of observed vs. predicted data, with R^2^ = 0.958 and n = 37.

When the accumulated disease severities observed in the four epidemics (AK-96, CL-12, CL-13, and CL-14; Figure 6, Figure 7, Figure 8 and Figure 9) were compared to the accumulated infection severities predicted by the model, the concordance correlation coefficient was CCC = 0.975, the root mean square error was RMSE = 0.061, and the coefficient of residual mass was CRM = 0.016 (Figure 10). Overall, these statistics indicated a good fit of observed and predicted data.

## 3. Discussion

In this research, a mechanistic model was developed that represents a “system”, i.e., epidemics caused by *Colletotrichum* spp. on grape clusters. In plant disease modeling, a system is a well-defined segment of reality, separated from the outside environment by clear boundaries [50]. Here, the system is composed of a single grape plant surrounded by similar grape plants in terms of size, growth, and development, and by the disease caused by *Colletotrichum* spp. on clusters under the “mean field” hypothesis [51]. According to this hypothesis, (i) the system and surrounding plants have the same structure and microclimatic conditions; (ii) there is a dynamic equilibrium in flows of conidia entering and exiting the system; and (iii) the probability of infection of any host site is everywhere the same within the system. The mean field hypothesis over space is shared by many epidemiological models [52,53]. The system in the current study encompasses the disease on clusters and disregards the disease on stems and leaves, which can also be affected [54]. This simplification affects the model in terms of the numbers of conidia that are produced and deposited on clusters or other plant parts. Preliminary evidence indicates that grape leaves artificially inoculated with conidia can support non-symptomatic infection [55] and that conidia can be produced on leaves by the conidia that germinate with phialides instead of germination tubes and by phialides that form on elongated germination tubes [56]. The role of these conidia in disease epidemiology has yet to be understood.

In developing the model, we first conducted a systematic literature review to obtain the current knowledge on the epidemiology of *Colletotrichum* spp. causing ripe rot of grapes and how that epidemiology is affected by weather and host plant conditions. However, a literature review provided incomplete information about some important aspects of the epidemiology, which forced us to make assumptions and to keep some variables constant [23]. The correctness of these assumptions was indirectly evaluated by comparing model predictions with independent data (data not used in model development): accurate predictions of real ripe rot epidemics would suggest that the assumptions were correct. For this purpose, we used disease data collected in 19 epidemics in three countries, which were characterized by diverse weather and cultivation conditions and a wide range of disease levels. This substantial range of conditions made our evaluation robust [23].

A first assumption of our model concerns the fungal species. Historically, *Colletotrichum gloeosporioides* and *C. acutatum* have been considered the main causal agents of ripe rot [2,5,6]. However, recent taxonomic revisions based on molecular analysis rearranged the genus, and both species are now considered to be species complexes [7,8,57]. In addition, other *Colletotrichim* spp. have been associated with the disease (as indicated in the Introduction). It follows that our knowledge about these pathogens is largely incomplete. For instance, we found studies on the production dynamics of primary inoculum and berry infection for *C. gloeosporioides* but not for *C. acutatum*; similarly, we found studies on conidia dispersal and flower infection for *C. acutatum* but not for *C. gloeosporioides*. We did not find epidemiological studies for the other *Colletotrichim* spp. In our model, we used the available data irrespective of the fungal species, even though differences among species might be expected. For instance, Greer et al. [5] found that *C. acutatum* forms appressoria and penetrates grape tissue faster than *C. gloeosporioides*. Similarly, species of *Colletotrichum* differ in splash dispersal efficiency, probably due to differences in mucilage matrix properties [58]. Even though we are aware that this assumption may lead to errors in model outputs, we assert that our model structure may be valid and adapted for the different *Colletotrichum* species causing ripe rot of grapes, since they share a fundamentally similar disease cycle [59]. As new species-specific epidemiological information becomes available, simple changes in model parameters may make the model output more precise.

The second assumption of our model concerns the dispersal of conidia. In *Colletotrichum*, conidia are usually produced in acervuli and are typically rain-splashed [59]. In acervuli, the conidia are embedded in a mucilagenous mass [60] that absorbs water and swells; during rainfall, the mucilage dissolves, and the conidial concentration in the suspension becomes diluted, which reduces the concentration of chemical inhibitors present in the mucilage, that prevent the germination of conidia [61]. Raindrops then produce splashes that disperse the conidia [62,63]. The only detailed data we found on the quantitative relationship between rain and conidial dispersal was in Madden et al. [63], who studied the effects of simulated rain intensity (2 to 60 mm/h) on the dispersal of *C. acutatum* conidia at various distances from the inoculum source represented by strawberry fruit: splashing droplets were collected on Petri plates with a selective medium, and the colony forming units were counted. Because the number of fungal colonies increased linearly with rain intensity >2 mm/h (colony number with 2 mm/h was close to 0), we assumed that the dispersal of conidia in the model is triggered by a rain that exceeds 2 mm/h. Comparison of model output with real epidemics, however, did not confirm this assumption; in 8 of 36 cases, disease growth was observed in the vineyard but was not predicted by the model because the rain intensity was below the threshold. This error mainly occurred (7 of the 8 cases) for disease observations during ripening, when the affected berries within clusters likely produced secondary conidia. This suggests the possibility that a light rain (<2 mm/h) can disperse conidia produced by acervuli in rotten berries to nearby berries within a cluster. Rajasab and Chawda [62] demonstrated that single drops (a drop was 3 mm in diameter and carried till 2000 conidia) were able to wash conidia from acervuli of *C. gloeosporioides* onto onion leaves. The washing-off of *Colletotrichum* conidia within an apple tree canopy was also demonstrated by Hamada et al. [64], and Guyot et al. [65] found that a light rain (a “drizzle”) can disperse *Colletotrichum* conidia within rubber trees. Daykin and Milholland [18] reported that the numerous insects attracted to rotting fruits may spread the secondary inoculum of *C. gloeosporioides*. Based on these findings, the model could be modified as follows: when secondary conidia are present on affected berries, any rain >0 mm/h can trigger dispersal. With this modification, we found that all of the missed infections were correctly predicted by the model (not shown). The model would clearly benefit from further studies on the dispersal patterns of *Colletotrichum* conidia in vineyards.

Because of a lack of knowledge, the following variables were kept constant in our model: (i) the time required after véraison for berries with quiescent infections to exhibit berry rot; (ii) the length of incubation in berries that are infected during ripening; and (iii) the latency period (LP) that determines when rotting berries begin to produce secondary conidia. These time periods may affect the impact model output, because they regulate the time when acervuli appear on the rotting fruit and therefore when secondary infection cycles begin. Considering that artificially inoculated berries kept at 27 °C and 100% RH showed symptoms in 2 or 3 days after inoculation [34] and that acervuli develop rapidly after the onset of ripe rot symptoms [5,33], we arbitrarily kept LP = 5 days. In cases when LP is shorter or longer than 5 days, the model could underestimate or overestimate, respectively, the effects of secondary infections. In our evaluations, however, the number of secondary infections predicted by the model and their severities were both significantly correlated with the disease incidence, indicating that the model provided a reliable representation of the secondary infection cycles. A better understanding of the time periods that regulate the epidemic dynamics is, however, desirable. Interestingly, for CL-14, the model predicted severe primary infections before véraison and no or light infections for the following 4 weeks, at which time disease symptoms appeared and disease severity subsequently increased (Figure 9). We suspect that this increase in disease severity might to some extent be sustained by the quiescent infections that progressively resulted in berry rot after véraison. Unfortunately, in the studies we found with the artificial inoculation of green berries, the disease was assessed at full ripening, and no information was provided on the time when these quiescent infections began to produce visible symptoms on berries [18,31,66].

A further simplification we made is that, in model evaluation, we did not consider the real dose k of primary conidia that can develop from overwintering inoculum sources (mummified berries, pedicels, and rachises). The real value of k in a vineyard may depend on many difficult-to-estimate factors, including the incidence and severity of affected clusters in the previous season and the proportion of them remaining on the trellis after harvesting and pruning. We therefore arbitrarily kept k = 1 as was previously done in models for downy and powdery mildews [22,25] and anthracnose [27]. For a precise parametrization of k, methods should be developed for the quantification of the primary inoculum of *Colletotrichum* in a vineyard. The results of model evaluation against real data, however, showed that the lack of this information did not affect the ability of the model to correctly predict infection events and their relative severity. This indicates that the current model can be considered useful for estimating which infections during the season may be more relevant than others, and that may therefore deserve special attention.

Overall, our model provided an accurate description of the system under investigation, as well as reliable predictions of infection periods and of disease progress during the season. The model, therefore, increases our understanding of ripe rot epidemics in vineyards. The model also provides directions for disease control.

Based on our simulations and evaluations, the primary inoculum affects disease progress for a long time during the season and is responsible for the infections occurring between flowering and véraison in the considered vineyards. This indicates that the reduction of inoculum sources such as the mummies left on the trellis and ground from the previous season is important for disease control. The control of primary infections at early growth stages of berries is also crucial. Once infected clusters begin to rot and produce conidia in the vineyard, the pathogen can rapidly spread to uninfected fruit in the same cluster or in neighboring clusters, and can thereby cause secondary infection cycles. Our model simulations confirmed that secondary infections significantly contributed to increases in the disease during ripening, and that secondary infections can spread within affected clusters via rain or drizzle [65] and insects [18]. Because the control of ripe rot under these conditions is often ineffective and may result in fungicide residue problems [67], growers should prevent the disease through timely fungicide applications, from flowering to véraison. In this context, our model can contribute to the scheduling of fungicides based on the risk of infection rather than on the calendar; scheduling fungicides according to the risk of infection results in an effective control of the downy and powdery mildew of grapevines with less use of chemicals [20,21] and, as a consequence, in a reduced risk of resistance to fungicides in *Colletotrichum* populations [68,69,70,71].

For the purpose of fungicide scheduling, our model can be considered an advancement compared to the forecasting system of Park et al. [29]. The latter system defines an infection risk (and recommends a fungicide application) when the temperature and wetness duration are favorable for the germination of ≥20% of conidia, as determined in laboratory assays [28]. In a field experiment in Chungha, Korea, in 1989 [29], the forecasting system suggested three fungicide applications, from flowering to harvest, which was one application fewer than the farmers’ conventional spray program. Both fungicide schedules provided only a partial disease control, indicating that they missed real infection events. We suspect that defining the risk of infection based on the occurrence of weather conditions conducive to the germination of 20% of conidia may be inadequate. For instance, this forecasting system may fail to detect infection events when the dose of available inoculum is high and the weather conditions allow for the germination of <20% of conidia. Unlike the forecasting system of Park et al. [29], our model considers all of the main epidemiological components leading to infection. Field experiments are now needed to clarify the advantages and disadvantages of the model-based fungicide scheduling relative to calendar-based fungicide scheduling.

## 4. Materials and Method

### 4.1. Literature Search

An extensive literature search was conducted to select studies that contain original data on the biology, ecology, or epidemiology of *Colletotrichum* spp. and its pathogenic interaction with *Vitis* spp. (i.e., the eligibility criteria). The literature search was carried out in the CAB Abstracts database (http://www.cabdirect.org, accessed on 5 May 2021), the Web of Science, Google Scholar, and CNKI (China National Knowledge Infrastructure). The following keywords were used in the literature search: (i) *Colletotrichum gloeosporioides* OR *Colletotrichum acutatum* OR the other species causing grape ripe rot (as listed in the Introduction section); (ii) ripe rot OR other common names; (iii) life cycle OR germination OR appressoria OR infection OR incubation OR latency OR latent period OR acervuli OR conidia OR spores OR pseudothecia OR perithecia OR ascospores OR perennation OR overwintering OR model OR prediction OR simulation. Each article found in the databases was first reviewed on the basis of the information in the title and abstract. If the article met the eligibility criteria, it was considered of potential interest; otherwise, it was discarded. Articles of potential interest were then retrieved and reviewed. Further articles were selected from the “References” section of the articles found; these articles were also retrieved and reviewed.

### 4.2. Systems Analysis and Model Development

Figure 1 shows the system under investigation: the life cycle of *Colletotrichum* spp. on grapes as deduced from the literature. Based on the grouping of Peres et al. [59] for the lifestyles of *C. acutatum* on hosts different from grapes, the lifestyle of *Colletotrichum* spp. on grapes can be defined as a hemibiotroph pathogen. Both *C. gloeosporioides* and *C. acutatum* overwinter mainly as dormant mycelium in mummified clusters (berries and infected pedicels) that remain in the vineyard from the previous season [4]. In the next season, acervuli produce abundant conidia splash-dispersed to healthy tissues and serve as inoculum for repeated primary infection cycles. Conidia germinate, produce appressoria, and penetrate the cuticle of berries, which are susceptible from flowering to ripening [4,17,32]. Infected berries, however, do not show symptoms until ripening, i.e., the infection remains quiescent until véraison or later [18,33]. During ripening, rotted berries can develop acervuli with conidia that serve as inoculum for secondary infections of other berries [4,16].

The relational diagram of the grape ripe rot system is shown in Figure 2, which uses systems analysis syntax [23,24]. State variables (boxes) that represent the stages of the system are defined. The flow from one stage to the next (arrows) is regulated by rates (valves) or switches (diamonds), which are in turn influenced by external and auxiliary variables (short segments and circles, respectively). The external variables include weather variables such as air temperature (T in °C), wetness duration (WD in h), and rainfall (R in mm). The rates are finally linked to external variables through mathematical equations (represented by dotted lines).

Mathematical equations linking external and auxiliary variables to rate variables were developed by using published data. Data on the pathogen or the disease and the influencing variables (e.g., temperature) were obtained directly from tables or graphs in the papers; the GetData Graph Digitizer 2.24 (http://getdata-graph-digitizer.com, accessed on 5 May 2021) was used to obtain precise data from published graphs. The data were then fit with mathematical equations, which were selected by using Origin 8 Pro (OriginLab Corporation, MicroCal, Northampton, MA, USA) and the Akaike information criterion [72]. The equation parameters were then estimated using the non-linear regression procedure of Origin 8 Pro. The magnitude of the standard errors of the model parameters, R^2^ adjusted for the degree of freedom, and the magnitude and distribution of residuals of predicted versus observed data were used to evaluate the goodness-of-fit.

### 4.3. Data for Model Evaluation

The model output was evaluated against field data retrieved from the literature, which covered 19 grape ripe rot epidemics that occurred between 1980 and 2014 in China, Japan, and the USA. The characteristics of the epidemics are summarized in Table 2; further details can be found in the original papers mentioned in Table 2. Table 2 also indicates the abbreviations (e.g., CH-80 and CL-12) used to indicate the location-year of the 19 epidemics.

In all of these epidemics, disease incidence was assessed as the percentage of affected clusters or berries in untreated plots when clusters were ripe and ready to be harvested. In some epidemics, severity was also assessed as the percentage of cluster surface affected by the disease. For CH-80 and CH-81, disease incidence was also repeatedly assessed during the season, from small green fruit to ripening. For CL-12, CL-13, and CL-14, the disease severity was assessed at weekly intervals between véraison and harvest (for 10, 12, and 9 weeks, respectively). For AK-96, six groups of clusters (each group containing 10 to 20 young clusters) were enclosed in paper bags in an affected vineyard. Each of these groups was exposed to the natural inoculum by removing the paper bags in the following periods: 2 to 8 July (corresponding to the fruit set); 8 to 15 July; 15 to 18 July; 18 to 26 July; 26 July to 2 August; and 2 to 4 August (berries still green). After each exposure period, the clusters were covered by paper bags again until berry maturity, when disease severity was assessed.

The weather data for running the model (i.e., hourly values of air temperature, relative humidity, wetness duration, and rainfall) were obtained from the authors upon request or were downloaded from the GSOD (Global Surface Summary of Day) database of NOAA (National Oceanic and Atmospheric Administration) for the weather station nearest to the experimental site indicated in the article. The approximate distance between the weather station and the experimental site was 100 m for CH, 2 km for CL, 12 km for BJ, and 19 km for AK. There were two papers of interest for model evaluation [73,74] for which we did not find weather data for running the model. 

Because the information on the amount of inoculum in each vineyard was not available to define the parameter k (seasonal number of conidia from overwintered primary sources) in the model, we kept k = 1 in all vineyards, so that the relative infection severity was expressed as the proportion of initial inoculum that caused the infection. The growth stages of vines were inferred from the articles.

### 4.4. Data Analysis

The model was evaluated for its ability to predict infection events and their relative severity. Based on the available data, we performed three kinds of evaluations, with the aim of answering the following questions: (i) Is there a relationship between the observed and predicted number of infection events and disease severity? (ii) Do the infection events predicted by the model during the season correspond to the disease observations in vineyards? and (iii) Is the model able to represent disease progress correctly?

#### 4.4.1. Predictions of Disease Incidence

Model predictions of the number of infection events (primary, secondary, and total) and their relative severities were compared with the disease incidence observed in the 19 epidemics at harvest or during the season (for CH-80 and CH-81), for a total of 37 observations (n = 37). Spearman’s correlation coefficient (ρ) was calculated between model predictions and observations. We used ρ, which is a nonparametric measure of rank correlation, because we were more concerned with whether the observed and predicted rankings of vineyards for disease incidence were similar rather than with whether the numerical output of the model was similar to the observed incidence. However, a regression analysis was conducted to estimate the relationship between the observed disease incidence (dependent variable) and the number of predicted infections (independent variable).

#### 4.4.2. Prediction of Infection Events

For CL-12, CL-13, and CL-14 (Table 2), the disease severity was assessed once per week for 10, 12, and 9 weeks, respectively. Each assessment date was categorized as O+ or O− based on whether or not an increase in disease severity was observed compared to the previous week. Similarly, each assessment was categorized as P+ or P− based on whether infections were predicted by the model in the time period −3 to −10 days, considering that no disease symptoms may appear in fewer than 3 to 4 days after infection. For AK-96 (Table 2), each of the six groups of clusters exposed to the natural inoculum for various periods between 2 July and 4 August was categorized as O+ or O− in case disease severity was =0 or >0, respectively, and P+ or P− in case the model predicted infections or not, respectively.

A contingency table (2 × 2) was prepared containing the true positive proportion (TPP or sensitivity, i.e., O+|P+), the true negative proportion (TNP or specificity, i.e., O−|P−), the false positive proportion (FPP, i.e., O−|P+), and the false negative proportion (FNP i.e., O+|P−).

#### 4.4.3. Prediction of Disease Progress

The disease severities observed in the four epidemics mentioned in the previous section (AK-96, CL-12, CL-13, and CL-14; Table 2) were compared to the accumulated infection severities predicted by the model by using linear regression analysis. To make the data comparable [75,76], we rescaled (from 0 to 1) each infection severity value to the disease severity observed in each epidemic at harvest. The goodness-of-fit of observed vs. predicted data was estimated by determining the root mean square error (RMSE), the coefficient of residual mass (CRM), and the concordance correlation coefficient (CCC) [77,78]. RMSE is the measure of the average distance between real data and the fitted line [78]. CRM represents the tendency of the model toward over- or underestimation; more specifically, a negative CRM indicates that the model overestimates, while a positive CRM indicates that the model underestimates [78]. CCC estimates the difference between the fitted line and the perfect agreement line; a CCC value of 1 indicates that the fitted line is identical to the perfect agreement line [77].

## Figures and Tables

**Table 1:** List of variables, rates, and parameters used in the model.

Abbreviation	Description	Unit
State variables		
S1	Conidia from overwintered primary sources	N per plant
S2	Conidia deposited on berries	N per plant
S3	Conidia causing infection in berries	N per plant
S4	Conidia leading to berry rot	N per plant
S5	Colonies in berries producing acervuli (i.e., fertile colonies)	N per plant
S6	Conidia produced on rotten berries	N per plant
Rate variables		
*DELR*	Delay rate at which quiescent infections become visible	0 to 1
*DEPR*	Deposition rate of conidia on flowers and berries	0 to 1
*INCR*	Rate at which infected berries become visible	0 to 1
*INFR*	Infection rate on flowers and berries	0 to 1
*LATR*	Rate at which infected berries become fertile	0 to 1
*MATR*	Rate at which primary conidia mature	0 to 1
*SPOR*	Sporulation rate on affected and fertile berries	0 to 1
Parameters, counters, and auxiliary variables		
k	Seasonal number of conidia from overwintered primary sources	N per plant
i	Counter of the day	N
j	Counter of the cohorts of primary conidia	N
t	Time required by quiescent infections to cause berry rot after véraison	days
GS	Growth stage of vines based on Lorenz et al. (1995)	0 to 99
IP	Incubation period	Days
LP	Latent period	Days
LA	Leaf area per plant	m^2^ leaf
BA	Berry area per plant	m^2^ berries
BLR	Berry to leaf area: BA/(BA+LA)	0 to 1
DD	Degree-days accumulated on moist days	°C day
Driving variables		
R	Hourly rainfall	mm
T	Hourly air temperature	°C
T_max_	Maximum temperature for sporulation	°C
T_min_	Minimum temperature for sporulation	°C
T_WD_	Mean temperature of the wet period	°C
WD	Duration of wet period	N hours

**Table 2:** Summary of the characteristics of the 19 ripe rot epidemics used for model evaluation ^1^.

Location (Literature Citation)	Year	Epidemic Abbreviation	Cultivar	Disease at Harvest	
				Incidence ^1^	Severity ^2^
Akita, Japan (Fukaya [31])	1986	AK-86	Campbell early	34.0	-^3^
	1987	AK-87		97.6	-
	1988	AK-88		66.7	-
	1989	AK-89		47.6	-
	1990	AK-90		75.4	-
	1991	AK-91		36.4	-
	1992	AK-92		56.0	-
	1993	AK-93		35.0	-
	1994	AK-94		55.2	-
	1995	AK-95		94.7	-
	1996	AK-96		52.0	-
	1997	AK-97		34.0	-
Castle Hayne, USA (Daykin and Miholland [18])	1980	CH-80	Carlos	53.8	-
	1981	CH-81		23.7	-
Beijing, China (Lv et al. [47])	2011	BJ-11	Merlot	29.0	-
Changli, China (Li et al. [48])	2009	CL-09	Cabernet Sauvignon	29.5	9.7
Changli, China (Pang [49])	2012	CL-12	Cabernet Sauvignon	25.8	17.3
	2013	CL-13		31.4	33.5
	2014	CL-14		26.1	29.4

^1^ Disease incidence was assessed as % of affected clusters (at AK-86 to AK-97, BJ-11, CL-09 to CL-14) or berries (at CH-80 and CH-81). ^2^ Disease severity was assessed as the percentage of cluster surface affected by the disease. ^3^ Severity data not available

## Data Availability

Data is contained within the article and Supplementary Materials.

## Supplementary Materials

The following are available online at https://www.mdpi.com/article/10.3390/plants10112288/s1. Figure S1. Cumulative proportion of mature conidia of Colletotrichum gloeosporioides produced by overwintered inoculum sources as a function of degree-days (DD) accumulated after bud break of vines when temperature was >5 °C and rain was >1 mm. Dots show the average data from Fukaya [1] and Daykin and Miholland [2] in steps of 50 degree-days, and whiskers are standard errors; the curve shows the fit of the data with Equation (1) in the main text, with R² = 0.81. Figure S2. Relationship between berry area per plant (m2, calculated based on the example provided in the text) and the time after early flowering (days); GS = 60 is the growth stage of vines based on Lorenz et al. [7]. Figure S3. Relationships between temperature (°C) and rescaled infection severity by Colletotrichum acutatum (data of Steel et al. [8]), and C. gloeosporioides (data of Yun and Park [9]). Dots show the average data from the mentioned papers, and whiskers are standard errors; the curve shows the fit of the data by Equation (4) in the main text, with R² = 0.88. Figure S4. Relationship between wetness duration (hours) and rescaled infection severity by Colletotrichum acutatum (data of Greer et al. [10]) and by C. gloeosporioides (data of Yun and Park [9]). Dots show the average data from the mentioned papers, and whiskers are standard errors; the curve shows the fit of these data by Equation (5) in the main text, with R² = 0.95. Figure S5. Relationships between temperature (°C) and rescaled production of conidia by Colletotrichum acutatum and C. gloeosporioides. Dots show the average data from the literature mentioned in the text, and whiskers are standard errors; the curve shows the fit of the data by Equation (9) in the main text, with R² = 0.83.
