# Supplementary material for: Modeling the Effects of the Environment and the Host Plant on the Ripe Rot of Grapes, Caused by the Colletotrichum Species
Source: Plants (Basel). 2021 Oct 25;10(11):2288. doi: 10.3390/plants10112288 (PMC8623558; doi:10.3390/plants10112288)
Supplement: Supplementary file 1 [file plants-10-02288-s001.zip › SM (to Plants).pdf]

SUPPLEMENTARY MATERIAL

Details on model equations

<u>*Maturation of primary conidia [equation (1)]*</u>

For the development of equation (1), we used data from Fukaya [1] and Daykin and Miholland [2]. Fukaya [1] sampled rain-borne conidia by placing a funnel trap under affected peduncles in a vineyard in Akita Prefecture (Japan) from 1986 to 1997. Daykin and Milholland [2] collected conidia from affected mummies and pedicels by using a similar method in a vineyard in Castle Hayne (NC, USA) from 1980 to 1982; we used the data of 1980 only, because those of 1981 and 1982 were biased by the presence of secondary inoculum. Both papers showed the numbers of conidia in samples over time, with time expressed as calendar days.

We expressed the cumulative numbers of conidia sampled in each season on a 0 to 1 scale, where 1 is the total of the conidia sampled in a season, and used the rescaled data for equation fitting (Figure S1). We also expressed the time after bud break as cumulative degree-days by accumulating daily temperature when T > 5°C (base temperature) and rain > 1 mm (base rain). We defined the base temperature according to Wang et al. [3], who reported no fungal growth and no sporulation on PDA at 5°C. We accumulated temperature only on rainy days, because conidia were produced during rainy periods in the spring [4]; we defined 1 mm of rain as a threshold based on the best fit we obtained in a preliminary analysis (*not shown*).

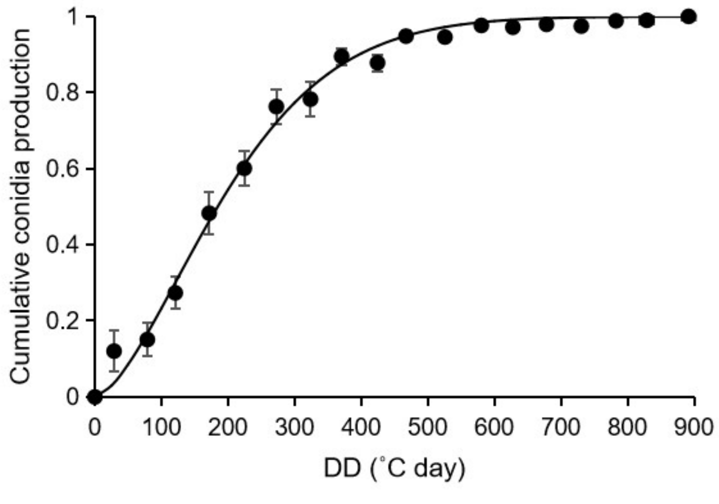

Figure S1. Cumulative proportion of mature conidia of *Colletotrichum gloeosporioides* produced by overwintered inoculum sources as a function of degree-days (DD) accumulated after bud break of vines when temperature was > 5°C and rain was > 1 mm. Dots show the average data from Fukaya [1] and Daykin and Miholland [2] in steps of 50 degree-days, and whiskers are standard errors; the curve shows the fit of the data with equation (1) in the main text, with R^2=0.81.

Rain and spore dispersal

The model assumes that the dispersal of conidia from acervuli is triggered by a rain > 2 mm/h, which is defined based on the spore dispersal experiment of Madden et al. [5]. In the latter experiment, the effect of rain intensity (millimeters per hour) on splash dispersal of *Colletotrichum acutatum* conidia from infected strawberry fruit was determined under simulated rains with intensities of 2 to 60 mm/h. Spore dispersal was assessed by collecting the splashing droplets with conidia in gravity samplers, consisting of Petri plates with a selective medium, and then counting the colony forming units. Colony numbers declined with the distance from the source, increased over time to a maximum, and then declined. Total colonies increased linearly with rain intensity when rain intensity was > 2 mm/h.

Berry-to-leaf ratio

The size of the system is defined by LA, the leaf area of the reference grapevine plant of the model system at flowering and after, and by BA, the berry area of this plant. The berry-to-leaf area (BLR) can be easily calculated as BA/(BA+LA).

For example, for *Vitis vinifera* L. cv. Barbera planted with a 2.5 m between-row spacing and a 0.9 m within-row spacing in a single Guyot system in northern Italy [6], the maximum leaf area of a grapevine plant at flowering and after was 3 m^2, the number of clusters per vine was 20, the numbers of berries per cluster was 85, each cluster having a maximum surface area of 531 mm^2 (radius = 0.6 mm) at late ripening. Therefore, the maximum value of BA in our model is 0.9 m^2 per plant (= 0.531/1000000 m^2 per berry times 85 berries per cluster times 20 clusters per plant), and the maximum berry-to-leaf ratio is BLR = BA/(BA+LA) = 0.9/(0.9+3.0) = 0.231. Obviously, BA increases from the start of flowering to full ripening based on the example of Figure S2.

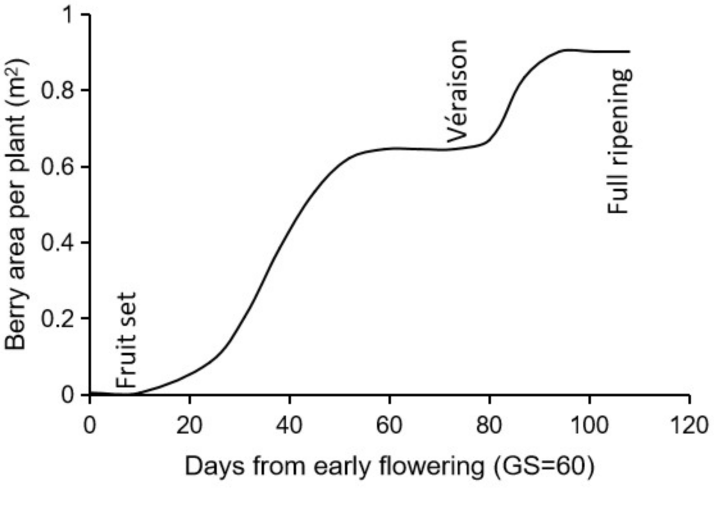

Figure S2. Relationship between berry area per plant (m^2, calculated based on the example provided in the text) and the time after early flowering (days); GS = 60 is the growth stage of vines based on Lorenz et al. [7].

Infection [equation (4)]

For the development of equation (4), we used data from Steel et al. [8] and Yun and Park [9]. To study the effect of temperature on infection of flowers, Steel et al. [8] immersed detached inflorescences of *V. vinifera* (cv. Chardonnay) in a suspension of *Colletotrichum acutatum* conidia and incubated them at 15, 20, 25, or 30°C for 24 h. Yun and Park [9] investigated the effects of temperature (10, 15, 20, 25, and 30°C) and wetness duration (0, 2, 4, 8, 12, 16, 20, and 24 h) on the formation of appressoria by germ tubes developed by the conidia of *C. gloeosporioides* that were artificially inoculated on grape berries of Riesling and Seibel 9110; to be consistent with the data of Steel et al. [8], we only used the 24-h data of Yun and Park [9].

The original disease data were expressed as % incidence of affected flowers and % of conidia that formed an appressorium on grape berries, respectively; we rescaled these data relative to the maximum value observed in each experiment on a 0 to 1 scale, and used the rescaled data for equation fitting (Figure S3).

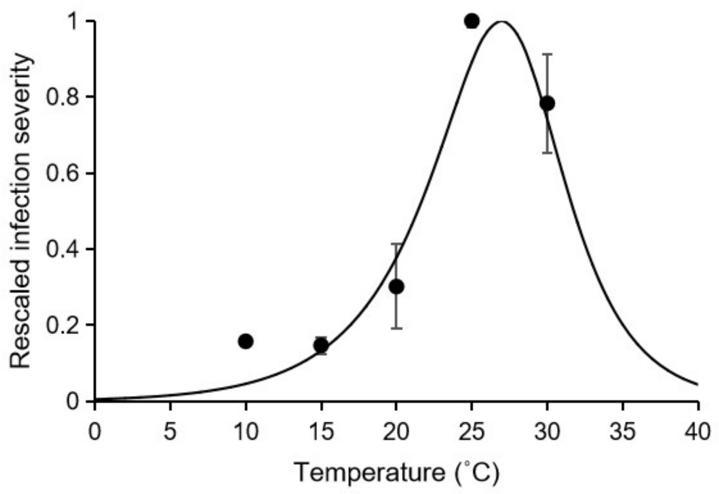

Figure S3. Relationships between temperature (°C) and rescaled infection severity by *Colletotrichum acutatum* (data of Steel et al. [8]), and *C. gloeosporioides* (data of Yun and Park [9]). Dots show the average data from the mentioned papers, and whiskers are standard errors; the curve shows the fit of the data by equation (4) in the main text, with R^2=0.88.

Infection [equation (5)]

For the development of equation (5), we used data from Greer et al. [10] and Yun and Park [9]. Greer et al. [10] studied the influence of wetness duration on flower infection by immersing detached inflorescences of *V. vinifera* (cv. Chardonnay) in a conidial suspension of *Colletotrichum acutatum* and incubating them at 25°C for 2, 4, 6, 12, 18, and 24 h. As indicated in the previous section, Yun and Park [9] investigated the effect of temperature (10, 15, 20, 25, and 30°C) and wetness duration (0, 2, 4, 8, 12, 16, 20, and 24 h) on the formation of appressoria by germ tubes developed by the conidia of *C. gloeosporioides* that were artificially inoculated on grape berries of Riesling and Seibel 9110; to be consistent with data of Greer et

al. [10], we only used the 25°C data of Yun and Park [9] for fitting the effect of wetness duration.

The original disease data were expressed as % incidence of affected flowers and % of conidia that formed an appressorium on grape berries, respectively; we rescaled these data relative to the maximum value observed in each experiment on a 0 to 1 scale, and used the rescaled data for equation fitting (Figure S4).

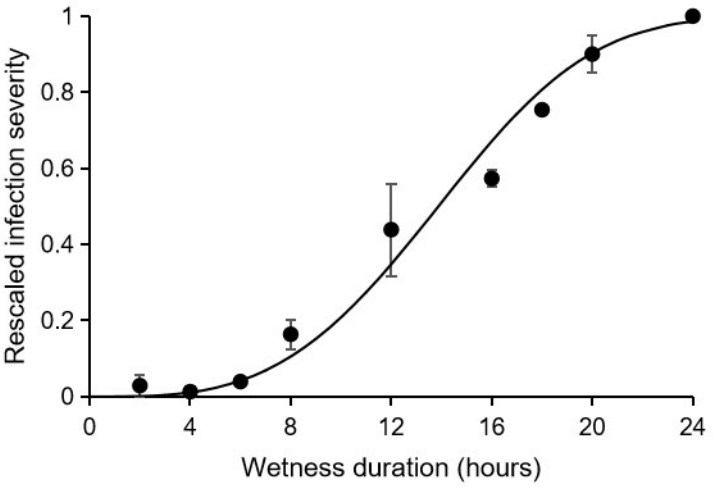

Figure S4. Relationship between wetness duration (hours) and rescaled infection severity by *Colletotrichum acutatum* (data of Greer et al. [10]) and by *C. gloeosporioides* (data of Yun and Park [9]). Dots show the average data from the mentioned papers, and whiskers are standard errors; the curve shows the fit of these data by equation (5) in the main text, with R^2=0.95.

Sporulation [equation (9)]

For the development of equation (9), we used data from Es-Soufi et al. [11], Everett et al. [12], Fernando et al. [13], King et al. [14], Liu et al. [15], Fitzell and Peak [16], Mello et al. [17], Pandey et al. [18], Wastie [19], Wang et al. [3], and Veloso et al. [20].

Es-Soufi et al. [11] determined the effect of temperature on the production of conidia by *Colletotrichum acutatum* on PDA at different temperatures (5, 18, 23, 25, 27, 30, and 37°C); Fernando et al. [13] and Liu et al. [15] did similar experiments on PDA at temperatures between 5 and 40°C (at 5°C intervals) and at 6, 8, 12, 14, 16, 18, 20, 24, 26, 28, 32, 34, and 36°C, respectively. Mello et al. [17] plated *C. gloeosporioides* from green pepper on PDA, incubated the plates at 15 to 35°C (at 5°C intervals), and finally enumerated the conidia at 7 and 12 days after plating. Wastie [19] studied the effect of temperature (15.0, 21.0, 26.5, and 32.0°C) on sporulation of *C. gloeosporioides* isolated from a rubber tree on Czapek-Dox agar. Wang et al. [3] studied the influence of temperature (5 to 40°C at 5°C intervals) on sporulation of *C. gloeosporioides* isolated from grape berries (cv. Kyoho) on PDA. Veloso et al. [20] confirmed that the maximum temperature for sporulation of *C. gloeosporioides* is 40°C by plating *C. gloeosporioides* isolated from a cashew plant on PDA.

Everett et al. [12] studied the effect of temperature (5 to 30°C, 5°C intervals) on the number of conidia produced by *C. acutatum* lesions on detached apples cv. Royal Gala. King et al. [14] studied the sporulation dynamics of *C. gloeosporioides* and *C. acutatum* in relation to temperature (5 to 35°C, 5°C intervals) on detached strawberry fruit. Fitzell and Peak [16] reported the effect of temperature (10.0, 12.5, 15.0, 17.5, 20.0, 22.2, 25.0, and 30.0°C) on the numbers of conidia produced by *C. gloeosporioides* on mango leaves. Pandey et al. [18] studied the effect of temperature on sporulation of *C. gloeosporioides* on guava fruits at 15, 20, 25, 30, 35, and 40°C and 75-80% RH for 10 days.

To combine the data extracted from the above experiments, we rescaled the original data relative to the maximum number of conidia found in each experiment on a 0 to 1 scale, and the averages of these rescaled values were used for equation fitting (Figure S5).

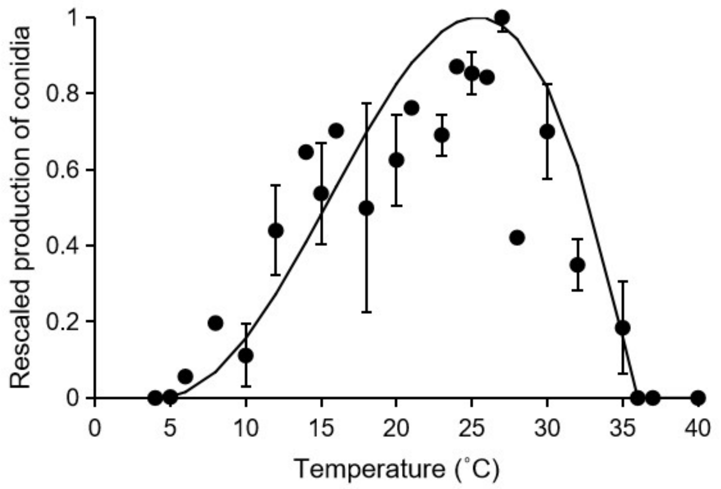

Figure S5. Relationships between temperature (°C) and rescaled production of conidia by *Colletotrichum acutatum* and *C. gloeosporioides*. Dots show the average data from the literature mentioned in the text, and whiskers are standard errors; the curve shows the fit of the data by equation (9) in the main text, with R^2=0.83.

References (in supplementary material)

1. Fukaya, M. Studies on etiology and control of grapevine ripe rot. I. Primary infection of grapevine ripe rot. *Bull. Akita Fruit-Tree Exp. Stn.* **2001**, *27*, 24–35.

2. Daykin, M.E.; Milholland, R.D. Ripe Rot of Muscadine Grape Caused by *Colletotrichum gloeosporioides* And Its Control. *Phytopathology* **1984**, *74*, 710–714.

3. Wang, P.S.; Liu, X.Q.; Wang, Y.Z.; Luan, B.H.; Zhang, W. Study on the Biological Characteristics of Grape ripe rot. *Jiangsu Agric. Sci.* **2009**, *01*, 128–129, doi:CNKI:SUN:JSNY.0.2009-01-051.

4. Milholland, R.D. Ripe rot. In *Compendium of Grape Diseases*; Pearson, R.C., Goheen, A.C., Eds.; American Phytopathological Society, St. Paul, MN, 1988; pp. 23–24.

5. Madden, L. V.; Yang, X.; Wilson, L.L. Effects of rain intensity on splash dispersal of Colletotrichum acutatum. *Phytopathology* **1996**, *86*, 864–874.

6. Bernizzoni, F.; Gatti, M.; Civardi, S.; Poni, S. Long-term performance of Barbera grown under different training systems and within-row vine spacings. *Am. J. Enol. Vitic.* **2009**, *60*, 339–348.

7. Lorenz, D.H.; Eichhorn, K.W.; Bleiholder, H.; Klose, R.; Meier, U.; Weber, E. Growth Stages of the Grapevine: Phenological growth stages of the grapevine (*Vitis vinifera* L. ssp. *vinifera*) - Codes and descriptions according to the extended BBCH scale. *Aust. J. Grape Wine Res.* **1995**, *1*, 100–103.

8. Steel, C.C.; Greer, L.A.; Savocchia, S. Grapevine inflorescences are susceptible to the bunch rot pathogens, *Greeneria uvicola* (bitter rot) and *Colletotrichum acutatum* (ripe rot). *Eur. J. Plant Pathol.* **2012**, *133*, 773–778.

9. Yun, S.C.; Park, E.W. Effects of temperature and wetness period on infection of grape by *Colletotrichum gloeosporioides*. *Korean J. Plant Pathol.* **1990**, *6*, 219–228.

10. Greer, L.A.; Harper, J.D.I.; Steel, C.C. Infection of *Vitis vinifera* (cv Chardonnay) Inflorescences by *Colletotrichum acutatum* and *Greeneria uvicola*. *J. Phytopathol.* **2014**, *162*, 407–410, doi:10.1111/jph.12201.

11. Es-Soufi, R.; El Kbiach, M.; Errabii, T.; Saidi, R.; Badoc, A.; Chaveriat, L.; Martin, P.; Lamarti, A. Biology and Physiology of *Colletotrichum acutatum* Strains Causing Strawberry's Anthracnose. *Agric. Sci.* **2018**, *09*, 974–990.

12. Everett, K.R.; Pushparajah, I.P.S.; Timudo, O.E.; Ah Chee, A.; Scheper, R.W.A.; Shaw, P.W.; Spiers, T.M.; Taylor, J.T.; Wallis, D.R.; Wood, P.N. Infection criteria, inoculum sources and splash dispersal pattern of *Colletotrichum acutatum* causing bitter rot of apple in New Zealand. *Eur. J. Plant Pathol.* **2018**, *152*, 367–383.

13. Fernando, T.H.P.S.; Jayasinghe, C.K.; Wijesundera, R.L.C. Factors affecting spore production, germination and viability of *Colletotrichum acutatum* isolates from *Hevea brasiliensis*. *Mycol. Res.* **2000**, *104*, 681–685.

14. King, W.T.; Madden, L. V.; Ellis, M.A.; Wilson, L.L. Effects of temperature on

sporulation and latent period of *Colletotrichum* spp. infecting strawberry fruit. *Plant Dis.* **1997**, *81*, 77–84.

15. Liu, A.Y.; Chen, W.X.; Gu, H.; Shi, J.Y.; Li, J. Biological characteristic of pathogenic fungus causing anthracnose of loquat fruit. *Acta Hortic.* **2007**, *750*, 465–447.

16. Fitzell, R.D.; Peak, C.M. The epidemiology of anthracnose disease of mango: inoculum sources, spore production and dispersal. *Ann. Appl. Biol.* **1984**, *104*, 53–59.

17. Mello, A.F.S.; Machado, A.C.Z.; Bedendo, I.P. Development of *Colletotrichum gloeosporioides* isolated from green pepper in different culture media, temperatures, and light regimes. *Sci. Agric.* **2004**, *61*, 542–544.

18. Pandey, R.R.; Arora, D.K.; Dubey, R.C. Effect of environmental conditions and inoculum density on infection of guava fruits by *Colletotrichum glososporioides*. *Mycopathologia* **1997**, *137*, 165–172.

19. Wastie, R.L. Secondary leaf fall of *Hevea brasiliensis*: factors affecting the production, germination and viability of spores of *Colletotrichum gloeosporioides*. *Ann. Appl. Biol.* **1972**, *72*, 273–282.

20. Veloso, J.S.; Lima, W.G.; Reis, A.; Doyle, V.P.; Michereff, S.J.; Câmara, M.P.S. Factors influencing biological traits and aggressiveness of *Colletotrichum* species associated with cashew anthracnose in Brazil. *Plant Pathol.* **2021**, *70*, 167–180.
